# BDNF and cognitive function in Alzheimer’s disease

**DOI:** 10.1192/j.eurpsy.2022.1673

**Published:** 2022-09-01

**Authors:** A. Sidenkova

**Affiliations:** Ural State Medical University, Psychiatry, Yekaterinburg, Russian Federation

**Keywords:** cognitive function in Alzheimer’s disease, bdnf, neurotrophic parameters, neuropsychological parameters

## Abstract

**Introduction:**

Alzheimer’s disease (AD) is a neurodegenerative pathology that develops mainly in elderly and senile people. Disruption of BDNF transport or suppression of its production appears to be typical for people of old age. Objective: To investigate the influence of Alzheimer’s disease on the secretion of brain factors and correlate with neuropsychological profiles.

**Objectives:**

12 men (2) and women (10) with Alzheimer’s disease were examined. The average age of the subjects was 76.25 + 4.89. Methods: MMSE, ADAS-COG, laboratory - BDNF was performed using the G7611 BDNF Emax (R) ImmunoAssaySystem 5 x 96 wells, BDNF Emax® Immunological test.

**Methods:**

2 patients have mild dementia, 8 patients have moderate dementia, 2 patients have severe dementia. The average age of patients with mild dementia was 72.0 + 1.0. The average MMSE score is 16.7 + 3.4.

**Results:**

Correlation analysis showed a close relationship between a pronounced decrease in memory in memory tests (ADAS-COG) and a pronounced decrease in blood BDNF content (r = 0.676). A close statistically significant relationship was found between a low result of the recognition test and a low blood BDNF content (r = 0.598).

**Conclusions:**

We assume that blood BDNF is a marker of pathologically accelerated aging of the central nervous system, since low test results for mnestic function are an indicator of severe degeneration in Alzheimer’s disease.

**Disclosure:**

No significant relationships.

